# Model Order Identification for Cable Force Estimation Using a Markov Chain Monte Carlo-Based Bayesian Approach

**DOI:** 10.3390/s18124187

**Published:** 2018-11-29

**Authors:** Shaodong Zhan, Zhi Li, Jianmin Hu, Yiping Liang, Guanglie Zhang

**Affiliations:** 1 College of Computer Science and Software Engineering, Shenzhen University, Shenzhen 51086, China; 2 Institute of Intelligence Cyber Sensing System, Shenzhen Academy of Robotics, Shenzhen 518057, China sdzhan@szarobots.com (S.Z.); jmhu@szarobots.com (J.H.); ypliang@szarobots.com (Y.L.)

**Keywords:** suspension bridge hanger cable, cable force estimation, model order identification, Bayesian approach, optimization

## Abstract

The tensile force on the hanger cables of a suspension bridge is an important indicator of the structural health of the bridge. Tensile force estimation methods based on the measured frequency of the hanger cable have been widely used. These methods empirically pre-determinate the corresponding model order of the measured frequency. However, because of the uncertain flexural rigidity, this empirical order determination method not only plays a limited role in high-order frequencies, but also hinders the online cable force estimation. Therefore, we propose a new method to automatically identify the corresponding model order of the measured frequency, which is based on a Markov chain Monte Carlo (MCMC)-based Bayesian approach. It solves the limitation of empirical determination in the case of large flexural rigidity. The tensile force and the flexural rigidity of cables can be calculated simultaneously using the proposed method. The feasibility of the proposed method is validated via a numerical study involving a finite element model that considers the flexural rigidity and via field application to a suspension bridge.

## 1. Introduction

As one of the primary load-bearing components of a cable system bridge, such as a cable-stayed bridge or a suspension bridge, the cable force should be adjusted during construction and monitored when the bridge is in service. Therefore, it is essential that the cable force is accurately measured. At present, the vibration method, according to which cable forces are estimated from the measured vibration frequency, is the most widely used method [1,2,3] for measuring cable forces due to its simplicity, speed, and economy relative to the method according to which cable forces are estimated via magneto-elastic permeability [4] or from a cutter ring containing strain gauges.

Currently, vibration methods that ignore cable sag are widely used to determine the tensile force of the hanger cables of a suspension bridge because the sag of vertical cables is so small as to hardly influence the measured frequency. The cable tension can be estimated based on taut string theory [5,6,7] or by established formulas for specified cables [8,9].

However, methods based on a single cable model are prone to errors when flexural rigidity values are large. Therefore, inverse analysis and system identification methods to estimate the hanger cable force based on finite element models (FEMs) have recently been proposed. These methods match the theoretical frequency from the numerical model with the measured frequency from an existing hanger cable. Park [10] first built a hanger cable FEM to obtain the modal frequency in an ideal geometric condition and then used the back analysis technique to optimize the variables using measured frequencies. Papadimitriou [11] discussed two different categories of model classified by different end plate connections and boundary conditions, using the Bayesian approach to estimate the cable force. Xie [12] used a genetic algorithm to optimize this inverse problem.

All of the aforementioned cable force estimation methods require the model order to be determined empirically, the accuracy of which depends on expert understanding of the structure. Generally, for measured frequencies of a low order, such as the first three orders, it is typically easy to specify the order from the similarity between the measured and theoretical frequencies from taut string theory or the interval between measured frequencies. However, in the case of high flexural rigidity, the empirical method is limited because of the sensitive effect of flexural rigidity, and a rapid increase occurs in the ratio between the measured frequency and higher-order theoretical frequencies [5,13]. In addition, in some cases, the frequency spectrum of cable vibration may indicate that low regions have a very small response or are submerged by noise frequency related to the low order, and that high regions have distinct dominant frequencies related to the high order. Hence, empirical determination plays a limited role in the case of high flexural rigidity, which hinders real-time cable force estimation.

To address this problem, we propose a new method for automatic model order identification that simultaneously estimates the cable force and the flexural rigidity of the hanger cable. The new method regards the model order as an unknown parameter, along with the flexural rigidity and cable force. This approach involves both continuous and discrete variables, and a stochastic method is adopted that can well-represent this sort of mixed mode. Here, a popular stochastic method, the Bayesian approach [14], is used. The feasibility of the proposed method is validated via two cases: a numerical study involving an FEM considering the flexural rigidity and a field application to a suspension bridge.

## 2. Dynamics of a Cable with the Flexural Rigidity

By ignoring cable sag, taking flexural rigidity into account, and having simply supported ends, the dynamic equation of a cable can be described as Equation (1) via the dynamic displacement of gravity direction v(x, t) at time t and length x [15]:

(1) $$N\frac{\partial^{2}v\left(x,t\right)}{\partial x^{2}}-EI\frac{\partial^{4}v\left(x,t\right)}{\partial x^{4}}=m\frac{\partial^{2}v\left(x,t\right)}{\partial t^{2}}$$

where N is the cable force, EI is the flexural rigidity, and m is the mass per unit length of the cable. For different conditions, various deformation equations can be derived from Equation (1) as a partial differential equation. Among them, Shimada [7] proposed a linear relationship (2) between ${\left(f_{k}/k\right)}^{2}$ and $k^{2}$, where *k* is the model order and $f_{k}$ is the *k-th* nature frequency, which is widely used for cable force estimation.

(2) $${\left(\frac{f_{k}}{k}\right)}^{2}=\left(\frac{1}{4mL^{2}}\right)N+\left(\frac{k^{2}\pi^{2}}{4mL^{4}}\right)EI$$

To scale the flexural stiffness effect on cables, a dimensionless parameter ξ is introduced, expressed as

(3) $$\xi =L\sqrt{\frac{N}{EI}}$$

## 3. Model Order Identification

In the proposed method, the model order is regarded as an unknown parameter in the cable vibration model. In addition, this vibration model has another two unknown parameters—cable force and flexural rigidity. The input is multiple measured frequencies extracted from the frequency spectrum. The range of model order is limited considering a completely slack cable. Finally, the three parameters are optimized through Bayesian estimation. However, an integral is required for each item of the Bayesian formula. We need to balance the number of integral points, as insufficiency will result in an obscure or erroneous output, and excessive data will severely prolong the computational time. The MCMC method can be used to complete this integral task. For a random sampling point (parameter vector), the MCMC method determines acceptance or rejection, and another point is resampled based on the previously accepted point. The Bayesian approach is a feature of the acceptance mechanism.

### 3.1. Frequency-Domain Analysis

Measured frequencies of the cable are determined by identifying the peaks from the power spectrum (PS) [1]. Researchers often use fast Fourier transform (FFT) in PS implementation. However, the calculation of Fourier spectra overlooks that a signal is a sample of a stochastic process; as such, much of the signal content is merely noise. Thus, it is a completely different task to obtain spectra from a theoretical signal versus a real cable vibration signal, the latter of which involves a stochastic process. Recently, output-only system identification for modal analysis, such as frequency domain decomposition (FDD) [16] and stochastic space identification (SSI) [17], has received considerable attention. Compared with the single FFT, these methods have the advantage of the PS being more distinguishable when the test structure is excited by ambient forces.

Here, FDD is adopted for frequency extraction. The power spectral density matrix of outputs of the structure can be defined as a matrix $G_{yy}\left(j\omega \right)$ as discrete frequencies $\omega =\omega_{i}$. Then, to obtain the measured frequencies, the power spectral density matrix in each frequency is decomposed by singular value decomposition, i.e., $G_{yy}\left(j\omega_{i}\right)=\;U_{i}S_{i}U_{i}{}^{H}$, where matrix $U_{i}=\left[u_{i1},u_{i2},\ldots ,u_{im}\right]$ contains the singular values $u_{ij}$, and matrix $S_{i}$ contains the singular values $s_{ij}$. The dominant peaks of these singular values belong to the measured frequencies.

### 3.2. Model Order Range Limitation

Under the effect of flexural rigidity, the measured differs from the theoretical frequency obtained from taut string theory. This difference is significant if the flexural rigidity is high. As a result, it is challenging to confirm the order of measured frequencies. This section describes a method to limit the range of the model order and to provide optimal methods via the measured frequencies. An appropriate range of these orders must be provided. The measured frequency can be expressed further by the theoretical frequency value $\dot{f}$ from taut string theory given by

(4) $${\bar{f_{k}}}^{2}={\dot{f}}_{k}{}^{2}\left(1+C\right)$$

where $C={\left(\frac{k}{L}\right)}^{2}\frac{EI}{N}$. If the flexural rigidity is low, then it only minimally influences the measured frequency, i.e., the minimum value of the constant C is 0. Conversely, if the flexural rigidity is large, then the measured frequency is more strongly influenced by higher model orders for the same cable length. Consider a condition EI/N=β, where β represents a completely slack cable. Given a constraint $k\leq L/\sqrt{\beta }$, the range of C can be defined within 0≤ C≤1. Thus, the range of measured natural frequency is given as

(5) $${\bar{f_{k}}}^{2}\leq 2{\dot{f}}_{j}{}^{2}$$

where $2{\dot{f}}_{j}{}^{2}$ represents the nearest values to ${\bar{f_{k}}}^{2}$. Because of the sequence, the measured frequencies should not share the same order; moreover, measured frequencies of high value should have a higher order than those of low value. Finally, for a set of measured frequencies with number *s*, the range of the order of the *s-th* measured frequency $k_{s}$ can be defined as

(6) $$\{\begin{array}{c} j+1\leq k\leq L/\sqrt{\beta }\\ k_{s}<k_{s+1}\; \end{array}\;\left(k\in Z\right)$$

### 3.3. Bayesian Parameter Estimation

After several measured frequencies are extracted from the PS, a parameterized model is built as follows:

(7) $${\bar{f_{k_{s}}}}^{2}-\frac{k_{i}{}^{2}}{4mL^{2}}N-\frac{k_{i}{}^{4}\pi^{2}}{4mL^{4}}EI=\varepsilon_{i}$$

where $\bar{f_{k_{s}}}$ is the *s-th* measured frequency, for which the range of the order $k_{i}$ is defined in the last section, and $\varepsilon_{i}$ is the *i-th* error value. The Euclidean norm of $\varepsilon_{i}$ is minimized to invert the measured frequency for the model parameters (model order, cable force, and flexural rigidity). This inversion problem can be solved using a deterministic method, such as the Gauss-Newton method, or using a stochastic method, such as the Bayesian approach. Because of the nonlinearity of the forward modeling and the general non-uniqueness and ill-posed nature of the inversion problem [18], many local optimal solutions may exist in this high-dimensional inversion problem. Relative to the deterministic method, the stochastic method provides a global solution and a superior approach to quantify uncertainty in the inversion problem. More importantly, the stochastic method has the capacity to represent mixed models with continuous and discrete variables. Therefore, a stochastic method—the Bayesian approach—is adopted here.

The Bayesian approach is based on Bayes’ theorem. This theorem states that, given the observed data, the probability of the model (also called the posterior probability function) depends on the likelihood function, the prior distribution, and the evidence. The Bayesian approach has been applied in many fields and has been proved to be a powerful approach to solving high-dimension inversion problems, such as finite-element-model updating [19,20,21,22] and artificial neural networks. The fundamental rule that governs the Bayesian approach is described as

(8) $$P(\theta |D)=\frac{P(D|\theta )P\left(\theta \right)}{P\left(D\right)}$$

where θ is a vector of the updating parameters; P(θ) is the probability distribution function of the updating parameters in the absence of any data, which is known as the prior distribution; *D* is the model output; P(D) is a normalization factor; and P(D|θ) is the likelihood probability distribution function. In addition, the quantity P(θ|D) is the posterior probability distribution function following a set of model outputs.

In this study, given the number of measured frequencies *s*, the vector of updating parameters θ is formed as $\theta =\left\{k_{1},\ldots ,k_{s},N,EI\right\}$. The prior distribution function P(θ) reflects prior information, which is defined as

(9) $$P\left(\theta \right)=\overset{s+2}{\underset{i=1}{\prod }}P\left(\theta_{i}\right)$$

In neural networks, the Gaussian prior has been successfully used to identify a large unknown parameter between two nodes, also known as weight; therefore, we assume this prior can successfully be used to identify a small number of parameters in this study. However, we find that the uniform distribution is more appropriate for the prior distribution of the model order, perhaps due to the integer property and sparse values of the model order. Studies have considered the solution for the prior distribution of integer values [15,23] in a linear model. In this study, the method described by [24] is used with the value range decided by Equation (9). The likelihood probability distribution function P(D|θ) is defined as the sum of squares of the error vector and is written as

(10) $$P(D|\theta )=\frac{1}{Z_{D}}exp\left(-\overset{s}{\underset{i=1}{\sum }}\varepsilon_{i}{}^{2}\right)$$

where $Z_{D}=\int exp\left(-\overset{n}{\underset{i=1}{\sum }}\varepsilon_{i}{}^{2}\right)d\theta$. The posterior distribution is still proportional to the numerator of Equation (8) after ignoring the normalization factor P(D).

### 3.4. Markov Chain Monte Carlo (MCMC) Method

It is apparent that both the prior distribution and the likelihood probability distribution are based on integral calculation. Due to the relatively high dimension of the updating parameter vector, it is challenging to solve the posterior distribution analytically. Thus, an MCMC method is used to avoid multi-dimensional integration by the sampling based on the posterior distribution.

**Figure 1:**
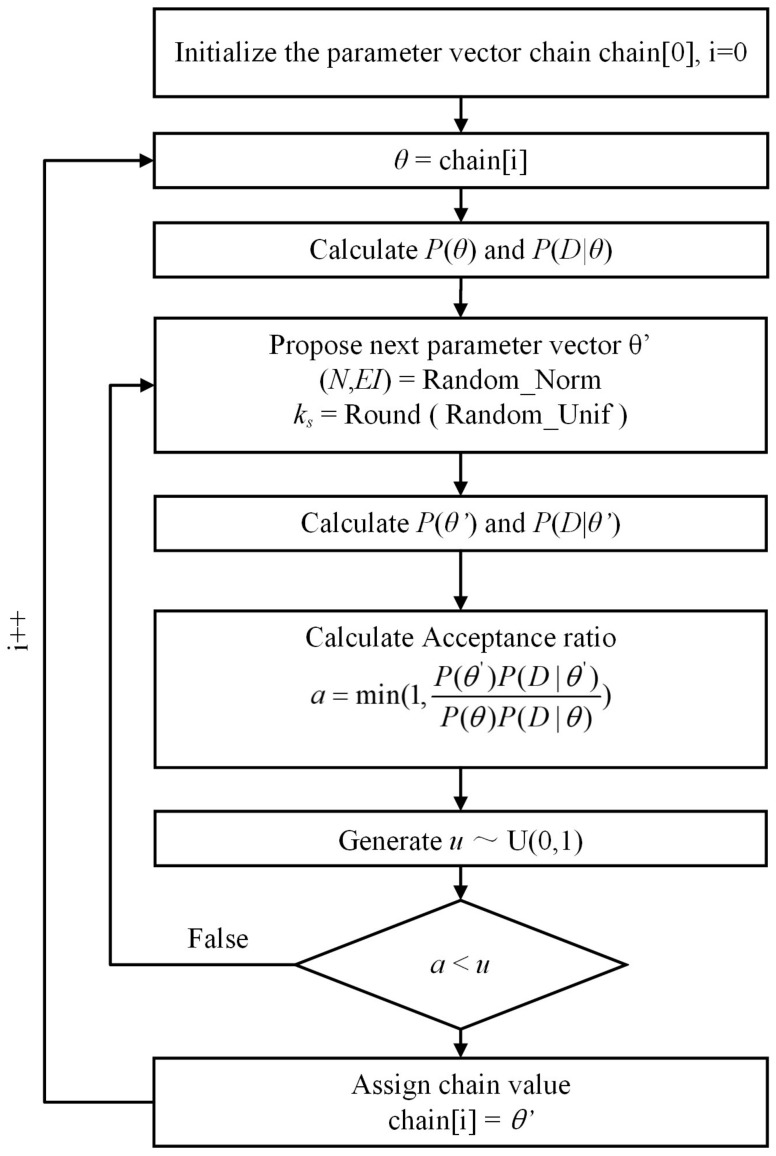
M-H algorithm for MCMC sampling.

MCMC methods belong to a class of algorithms for sampling from a probability distribution. These methods are based on building a Markov chain that has the desired distribution as its equilibrium distribution. After multiple iterations using the specific accept/rejection rule, the state of the chain is used as a sample of the desired distribution. Considering that the conditional distributions of some components in this study are nonstandard [25], the Metropolis-Hastings (M-H) algorithm is used as the sampler. Step-by-step instructions for the MH algorithm used in this study are presented in Figure 1.

Note that the prior distribution of the model order is the uniform distribution when the prior distribution of other updating parameters is the normal distribution. Because the acceptance ratio is related to the comparison between the posterior distribution of the proposal parameter vector and the next predicted parameter vector, considering the normalization factor is a constant, the normalization factor is ignored and does not require calculation.

In this study, we select a Gelman-Rubin diagnostic [26] to monitor the convergence of MCMCs. The Gelman-Rubin diagnostic is a general approach to monitoring the convergence of MCMC multi-chain outputs with starting values that are over-dispersed relative to the posterior distribution. The diagnostic assumes normality of the marginal posterior distributions. Convergence is assessed by comparing the estimated between-chains and within-chain variances for each model parameter. Large differences between these variances indicate non-convergence.

## 4. Feasibility Validation

### 4.1. Numerical Study

**Figure 2:**
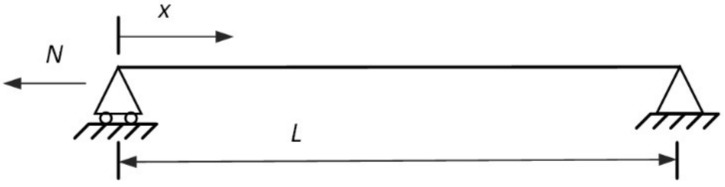
Cable model with hinged ends.

This section verifies our proposed method by judging whether the known parameters (model order, cable force, and flexural rigidity) are equal to the solutions for the frequencies from a simulated cable. Consider a cable without sag and with both ends hinged, as shown in Figure 2. The cable prototype is a hanger cable described previously [27]. Its mass per unit length is 13.6 kg/m and the diameter of the cross section is 56 mm. Four cables with different flexural rigidity values are used, with flexural rigidities of 63%, 158%, 317%, and 31% of the ideal value (Table 1). A linear FEM of the cable is programmed in MATLAB for eigenvalue analyses. The measured frequencies of the four cables are listed in Table 2. Four consecutive frequencies of each cable are selected to validate the proposed technique; these values correspond to the bold values in Table 2. The model order of the selected frequency is regarded as an unknown parameter. The four consecutive frequencies are matched with parameters of model order K1, K2, K3, and K4. Both the cable force and the flexural rigidity are initialized from 0.3, with an iteration number of 10,000. 

**Figure 3:**
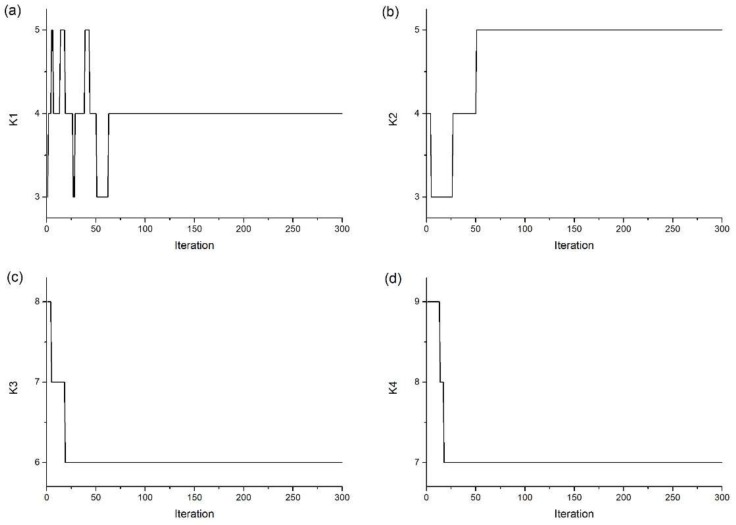
Convergence of the model orders (**a**) K1, (**b**) K2, (**c**) K3, and (**d**) K4 of Cable 1. K1, K2, K3, and K4 are assumed as unknown parameters which are related to the four consecutive frequencies from the simulated cable.

**Figure 4:**
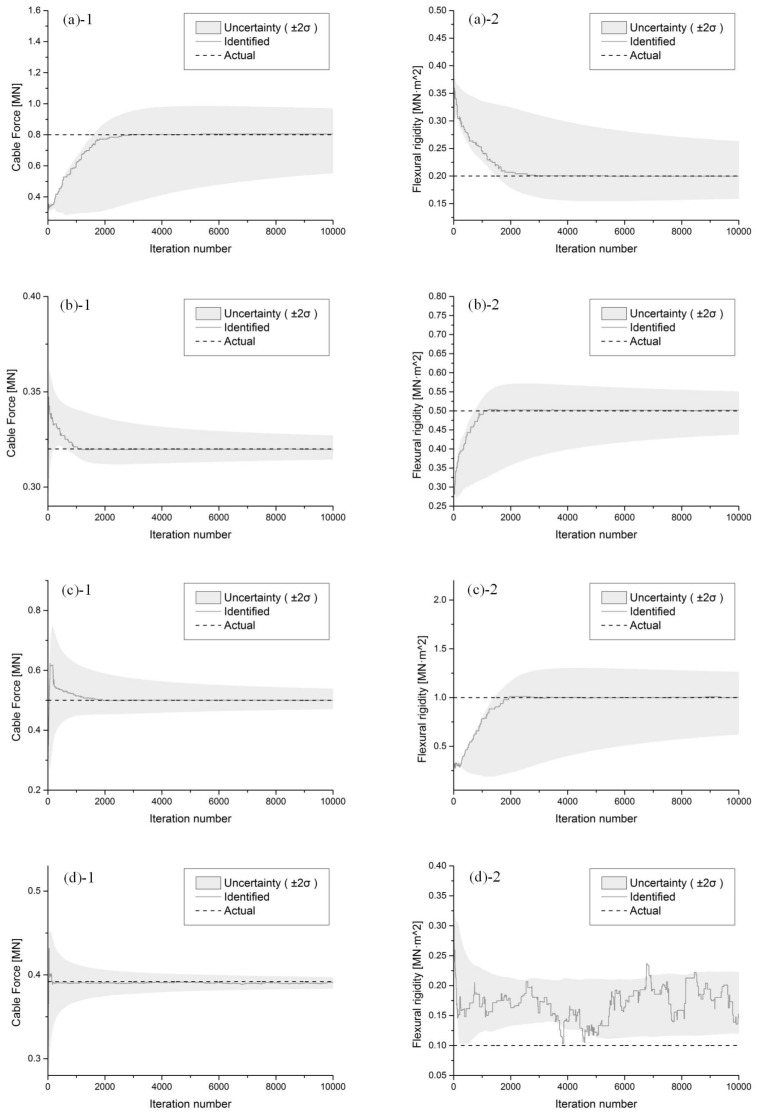
Convergence of the cable force and the flexural rigidity of (**a**) Cable 1, (**b**) Cable 2, (**c**) Cable 3, and (**d**) Cable 4.

Figure 3 shows the convergence of the model order of Cable 1. The convergence of the cable force and the flexural rigidity in the four cables are shown in Figure 4, demonstrating good agreement between the actual value under the ideal cable model and the ultimate converged value. Uncertainty in the estimation, expressed as the standard deviation (2*σ*), is denoted by the gray shaded area along the corresponding curves. With the cable force and flexural rigidity approaching the true values, the estimation uncertainty tends to decrease as the accuracy of the estimation improves. However, for Cable 4, when the cable force approaches the true value, the flexural rigidity wanders between 0.1 and 2.5. This wandering is caused by the low level of flexural rigidity. When flexural rigidity is low, the natural frequency is influenced only minimally, thus allowing the identified flexural rigidity to take on values over a wide range.

Because of the integer property, the curve of the chain values is far from smooth. The model order ultimately converges to the actual value. Comparing the convergence of cable force and flexural rigidity in Cable 1 shown in Figure 4(a1,a2), the model order rapidly converges to a stable condition when the cable force and flexural rigidity require additional iteration counts to reach the converging lines. 

### 4.2. Field Application

**Figure 5:**
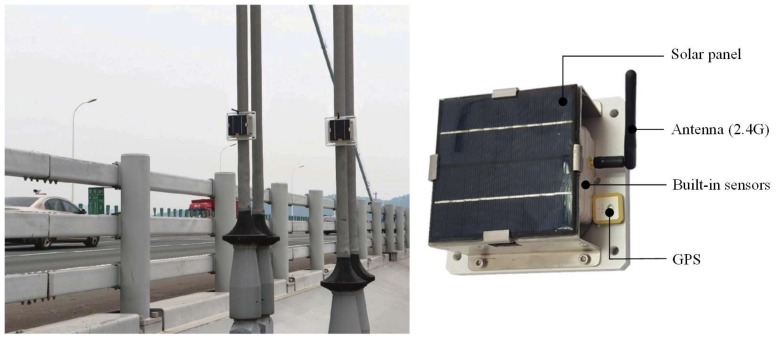
Wireless sensor nodes with two-axis MEMS accelerometers attached on hanger cables.

**Figure 6:**
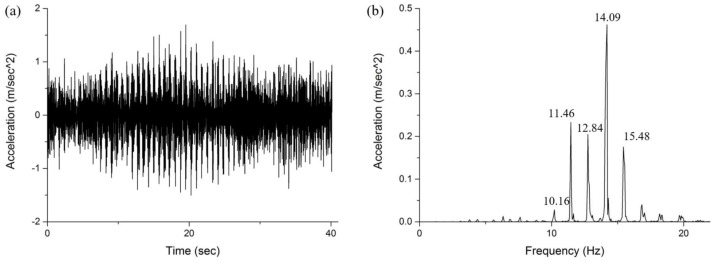
(**a**) Time history and (**b**) frequency spectrum of the acceleration response under ambient vibration.

The Huangpu suspension bridge, one of the 20 longest suspension bridges in the world, spans the main channel of the Pearl River, carrying the highway linking Caotang and Bicun in Guangzhou, China. It includes 144 hangers. To verify the proposed technique, we select hanger Cable N2 in the bridge as the study cable. Empirically estimating the model order is straightforward due to the low flexural rigidity of the cable. The properties of Cable N2 are shown in Table 3. Under an ambient vibration condition, we measure the acceleration time responses of the cable via the wireless sensor nodes with two-axis MEMS accelerometers attached along the longitudinal direction on the cable, as shown in Figure 5. The sensor nodes are wirelessly connected, have a sampling frequency of 100 Hz, and are synchronized via GPS timing. Figure 6a shows the acceleration responses of the cable under ambient vibration, and its PS is presented in Figure 6b.

**Figure 7:**
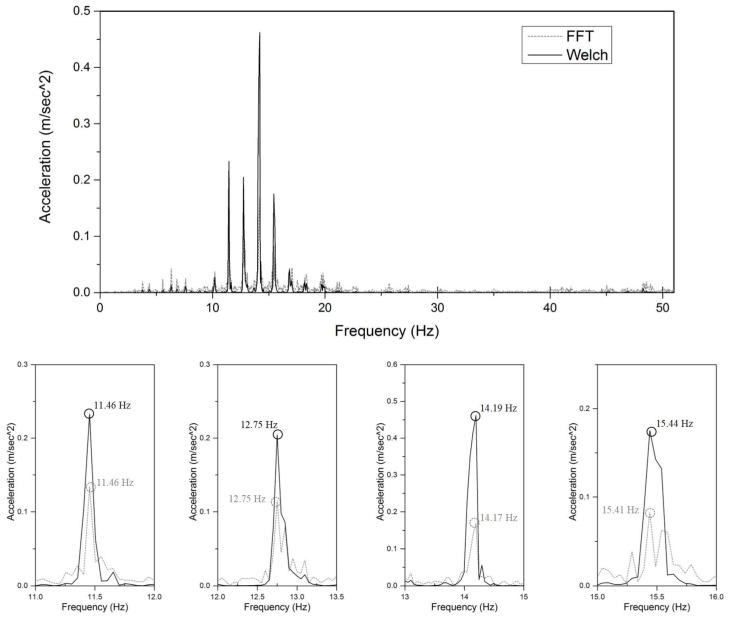
Difference between the single Fourier spectrum and the spectrum obtained using Welch’s method: (**a**) comparison of the whole spectra and (**b**) magnified sections.

FDD combined with Welch’s averaged and modified periodogram method is used to obtain the frequency spectrum. The window size for dividing the signal into sections is 1024, with an overlap number of 512. FDD can reduce the noise components in the frequency spectrum, as highlighted in Figure 7. The peak frequencies in the spectrum using FDD hold the same values as those in the single Fourier spectrum, whereas the sidelobes are decreased. Because of the large flexural rigidity, the interval between these frequencies is approximately regarded as an integral multiple of the first-order nature frequency. Thus, the model orders marked in Figure 6b are empirically determined to correspond to 16, 18, 20, 22, and 24. Next, these five frequencies are used to verify the proposed method, with orders 16, 18, 20, 22, and 24 associated with parameters K1, K2, K3, K4, and K5, respectively.

**Figure 8:**
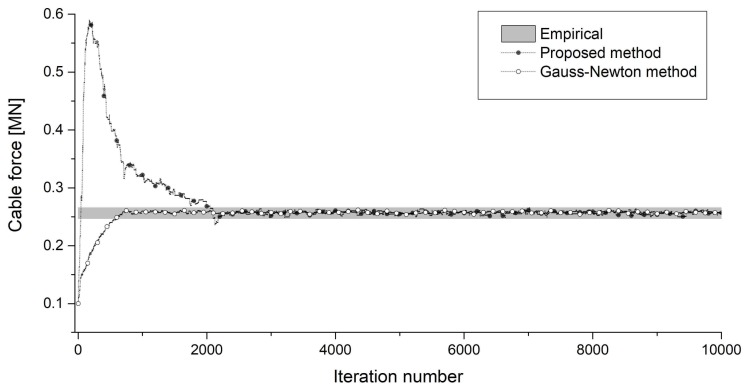
Cable force convergence in the proposed method without predefined model orders and the Gauss-Newton method with predefined orders.

**Figure 9:**
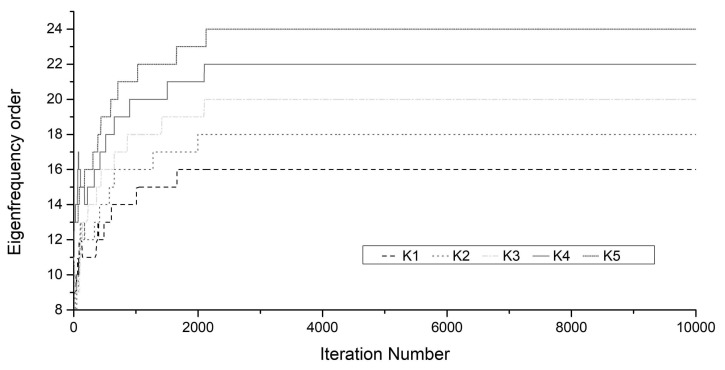
Convergence of the model order.

Figure 8 shows the convergence of cable force using the proposed method without predefining the model orders and the Gauss-Newton method with the predefined orders. Figure 9 shows the convergence of the five unknown model orders. The cable tensions estimated by taut string theory using peak frequencies of either weak or strong amplitude are represented by the green area, which is also based on the empirical predefined model order.

**Figure 10:**
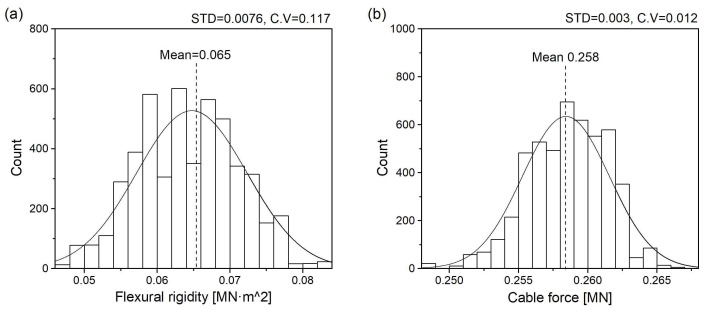
Histogram of the obtained samples of (**a**) flexural rigidity and (**b**) cable force.

According to Figure 8 and Figure 9, the proposed method can effectively estimate the cable tension while automatically identifying the order of measured frequencies. The Gauss-Newton method requires the pre-determination of an accurate model order; otherwise, the estimated cable force has a large error. The proposed method has a large error in the cable force at the beginning of the iteration process that is caused by the large range of model order. After many iterations, the cable force converges to the same value as in the Gauss-Newton method. Histograms of the obtained samples of cable force and flexural rigidity are presented in Figure 10. The mean values of cable force and flexural rigidity are 0.258 MN and 0.065 MN·m^2^ when the standard deviations are 0.0031 MN and 0.0076 MN·m^2^, respectively.

**Figure 11:**
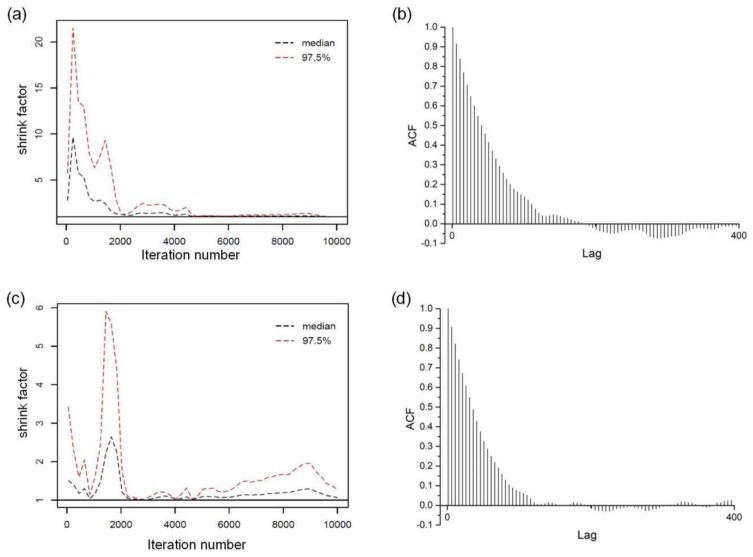
Shrink factor of (**a**) cable force and (**c**) flexural rigidity; with ACF of (**b**) cable force and (**d**) flexural rigidity in the last 5000 iterations.

The Gelman-Rubin diagnostic presents the scale reduction for each parameter by the shrink factor, as well as the auto-correlation. Figure 11 shows that the shrink factor of parameter *N* becomes stable after about 5000 iterations and wanders around 1 when the shrink factor of *EI* is less than 2. For both parameter *N* and *EI*, the plotted auto-correlation curve quickly decays to zero and then wanders around it. This indicates a good chain with a rapid rate and low degree of correlation between our samples, and also reveals that MCMC sampling has been done in an independent manner and the stationarity of the Markov chain has been attained. Based on the good performance for an iteration number of 10,000, we run MCMC for 10 and 20 times 10,000 again and find that the three runs have almost the same shape of histogram and convergence performance.

## 5. Conclusions

An automatic method for model order identification is proposed as an alternative to the existing method of cable force estimation, which uses empirical determination of the order of measured frequencies. The proposed method simultaneously calculates the cable force under the effect of flexural rigidity and includes the following features: (1) a measured frequency extraction procedure via Welch’s frequency spectrum, (2) a method to determine the range of the model orders of these frequencies, and (3) a Bayesian approach using these ranged orders as unknown parameters to determine both the cable force and the flexural rigidity. The Bayesian approach used here adopts an MCMC sampler, and the orders are given a uniform prior distribution. The feasibility of the proposed method is verified via a numerical study using an FEM model of cables and a field experiment, in which accelerometers are installed on hangers in a suspension bridge to collect the cable vibration data. The result of the field application shows that the proposed method can accurately calculate the model order and estimate the cable force, providing a value that is the same as the empirical value and the value obtained from the Gauss-Newton method. Note that the latter method must pre-define the model order, whereas the former method does not.

**Figure 12:**
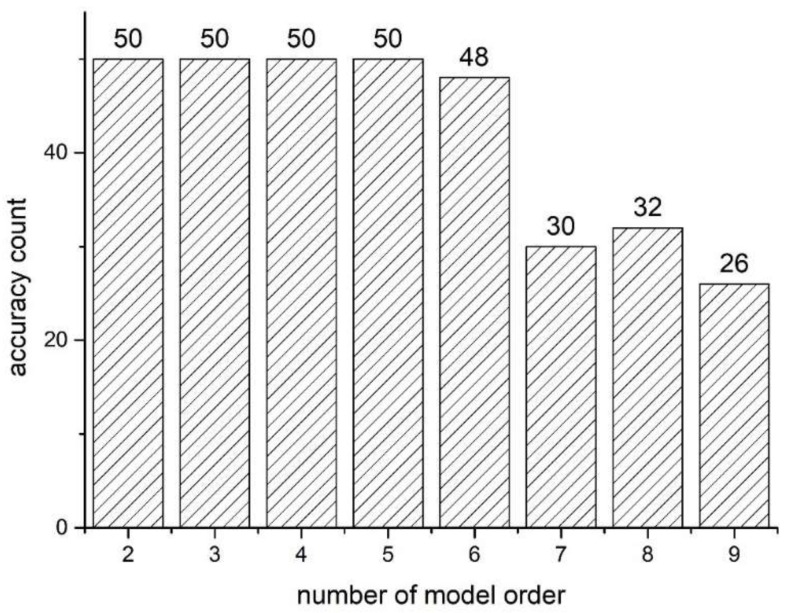
The accuracy performance for different numbers of model order.

The proposed method can also work on more or less model order frequencies. In the field application, we use five model orders as unknown parameters to investigate the capacity of the number of model orders in our proposed method, and we repeat the method 50 times for different numbers of model order. The result is shown in Figure 12. The proposed method works well when the number of the model order is less than six. However, the performance becomes worse with a greater number of model orders. It is difficult to find an appropriate indicator to show the effect of different initializations on the identification. So, for cable force and flexural rigidity, we use 100 Gaussian random values in the same orders of magnitude of the predicted value. For instance, if the cable force is predicted to be 0.2 MN, the initialized value can be set from 0.1 to 0.99. Four model orders are chosen and the values are randomly taken from the defined limited formula. The accuracy rate of the proposed method for this random initialization is 94%. 

## Figures and Tables

**Table 1:** Structural properties of four cables with no sag.

Cable No.	*ξ*	*N* (MN)	*EI* (×10^6^ N·m^2^)	*L* (m)
1	20.0	0.8	0.2	10
2	40.0	0.32	0.5	50
3	70.7	0.5	1	100
4	198.0	0.392	0.1	100

**Table 2:** Measured frequencies of the simulated cables.

Cable No.	Model Order							
	2	3	4	5	6	7	8	9
1	24.2	38.3	54.5 (K1)	73.4 (K2)	95.2 (K3)	120 (K4)	148.4	180
2	3	4.5 (K1)	6.1 (K2)	7.8 (K3)	9.7 (K4)	11.7	13.8	16.1
3	1.8	2.8	3.7	4.7	5.7 (K1)	6.7 (K2)	7.8 (K3)	8.8 (K4)
4	1.6 (K1)	2.4 (K2)	3.2 (K3)	4.1 (K4)	4.9	5.7	6.5	7.3

**Table 3:** Properties of the selected hanger cable.

Length (m)	Diameter (mm)	Elastic Modulus (Pa)	Theoretical Sectional Area (mm^2^)	Mass per Unit Length (kg/m)
110	56	1.1 × 10^11^	1490	13.6
